# National Football League Hand Injuries: Return to Sport and Performance

**DOI:** 10.7759/cureus.115825

**Published:** 2026-09-05

**Authors:** Madison Baldauf, Chloe Heiting, Gabriella Chin, George Economou, Priya Duvvuri, Carolina Herrera, Sruthi Katakam, Kate Nellans

**Affiliations:** 1 Orthopaedic Surgery, Eastern Virgina Medical School, Norfolk, USA; 2 Orthopaedic Surgery, Donald and Barbara Zucker School of Medicine at Hofstra/Northwell, Hempstead, USA; 3 Orthopaedic Surgery, Northwell Health, New Hyde Park, USA; 4 Orthopaedic Surgery, Rutgers New Jersey Medical School, Newark, USA

**Keywords:** finger fracture, football injury, hand fracture, national football league, orthopedic sports medicine, return-to-play, return to sport, sports performance, wrist fracture

## Abstract

Introduction

Injuries sustained by professional athletes can severely impact seasonal performance and future abilities. The aim of this study was to characterize the impact of phalanx, thumb, metacarpal, and carpal injuries on the performance of athletes in the National Football League (NFL). Analyzing outcomes for football players is significant because the functional demands of each position vary widely.

Methods

NFL players who sustained phalanx, thumb, metacarpal, or carpal fractures from the 2012-2013 to 2022-2023 seasons were identified using the public database Pro Sports Transactions. Performance data were extracted from Pro Football Reference, including player position and the number of games missed, as well as the number of games played, games started, and snap counts in the seasons prior to and following injury. Players were excluded from the study if additional injuries were sustained at the time of the hand/wrist injury or if insufficient data were available to confirm the injury. Descriptive statistics were performed to characterize the cohort. Statistical significance was defined as p < 0.05 and assessed using a single-factor ANOVA.

Results

A total of 304 players sustained a phalanx, thumb, metacarpal, or carpal injury, with three players having injuries in two of these categories: metacarpal, n = 110 (35.8%); carpal, n = 80 (26.1%); thumb, n = 78 (25.4%); and phalangeal, n = 39 (12.7%). Linebackers (14.6%) and wide receivers (12.8%) were found to be most likely to suffer one of these injuries. Most occurred during the preseason or in September, near the start of the regular season (p < 0.001). The average number of games missed due to an injury was 5.5 games; by injury, carpal: 6.4, phalangeal: 5.8, thumb: 5.4, and metacarpal: 4.3 (p = 0.02). Sixty-five players were injured in their rookie season and 58 in their final season. These players were excluded from further performance analysis. The average change from games played in the preceding season to those in the following season was +1.0, with no significant difference when stratified by injury. The average change in games started was +0.5 games, with thumb injuries having the most significant impact, as games started increased by an average of 2.0 games (p = 0.08). The overall average change in snap counts was +72.8; by injury, phalangeal: +120.1, carpal: +31.5, thumb: +125.7, and metacarpal: +50.0. Safeties had the greatest decrease in games played, with an average of 0.8 fewer, as well as the greatest decrease in games started, with an average of 2.0 fewer starts after injury. Safeties also had the largest decline in snap counts, with an average of 94.9 fewer snaps after injury.

Conclusions

Phalanx, thumb, metacarpal, and carpal fractures are common injuries in NFL players, with an average of about 25 recorded per season, and they have a measurable impact on player performance. Players missed an average of more than 5 games due to these injuries, which is nearly a third of a normal season. Fifty-eight players did not return to the NFL, suggesting that hand injuries may be costly to both players and organizations and should be carefully managed by trainers and medical staff. However, there was an overall improvement in performance among the players who returned the following season, which varied greatly depending on the position of the player. This study should help guide team physicians in anticipating the impact on players after a hand injury based on their position.

## Introduction

American football is among the most physically demanding contact sports, with injury rates that remain among the highest across all athletic disciplines [1]. The National Football League (NFL) subjects athletes to repetitive high-energy collisions, tackling, blocking, catching, and throwing that place the upper extremities at considerable risk. While lower extremity injuries have historically dominated the medical literature, upper extremity trauma represents a significant and underappreciated source of morbidity in professional football players [2,3].

Previous data from the NFL's injury surveillance database have demonstrated that upper extremity injuries are common, with 1,385 injuries documented over a 10-year period (1996-2005) [2]. This source also suggests that offensive and defensive linemen sustained the highest rates of hand injuries, with tackling identified as the mechanism responsible for the greatest proportion (28%) of these events [2]. Wrist injuries also account for a significant burden, with a mean of 27 days lost per injury [3].

Despite the frequency and functional impact of these injuries, current literature on hand injuries in the NFL remains quite limited, though recent studies have begun to characterize the detriment to performance. Sharareh et al. reported that the median return-to-play time following metacarpal fractures in NFL players was 15 days, with 71% of injured players missing time but returning within the same season [4]. Survey data from multiple surgeons across the NFL have revealed wide variability in management approaches for common hand injuries and time to return to play [5].

While return-to-play timelines have been increasingly reported, there remains a gap in the literature exploring athletic performance in NFL players after upper extremity injuries. Return to performance, however, has received very little attention thus far [6]. Changes in playing time and performance following injury are questions that remain largely unanswered.

The consequences of hand and wrist injuries may differ by position given the varying functional demands. For example, for certain players, including wide receivers and quarterbacks, grip strength and dexterity are essential for performance. For offensive and defensive linemen, the ability to sustain forceful wrist extension is fundamental. The purpose of this study is to investigate the epidemiology, return-to-play time, and post-injury performance following hand injuries in NFL players.

## Materials and methods

The Pro Sports Transactions database [7] was retrospectively analyzed for carpal, metacarpal, phalanx, and thumb injuries sustained by players in the NFL from the 2012-2013 to the 2022-2023 season. This was achieved by searching the terms "wrist," "hand," "finger," or "thumb" in the player search box and selecting "missed games due to injuries" and "movement to/from injured reserve" for the aforementioned dates. The date of injury or movement to injured reserve (IR) was recorded and verified using secondary sports-reporting websites.

Players were located in the Pro Football Reference database [8] to collect player position and return-to-sport data, which were extrapolated from the number of games missed due to the injury. The primary outcome was the impact on performance, which was analyzed by collecting the number of games played, number of games started, and snap counts from the pre-injury season to the post-injury season. Snap counts are a measure of the exact number of offensive and defensive plays for which the player was on the field during a given season. It was also noted if the injury occurred during the player’s rookie season or final season. These players were excluded from further analysis.

A total of 304 players who sustained carpal, metacarpal, phalanx, or thumb injuries were identified. The number of games missed due to injury was recorded for all players. This was defined as games in which the player had zero snaps recorded after the reported injury date. Confounding variables may include a player's decision to delay return to sport, outside factors, or additional injuries not addressed in the database. This was best handled by excluding players with multiple injuries reported or identified using a secondary sports-reporting website. Fifty-five players were injured in their rookie season and were excluded from further analysis due to the lack of pre-injury season data. Forty-eight players were injured during their final season and were excluded due to the lack of post-injury season data. Ten players sustained injuries in their only season in the NFL and were also excluded.

Descriptive statistics were used to characterize injury prevalence, position prevalence, and number of games missed. A single-factor ANOVA with a significance level of α = 0.05 was used to compare games missed and performance metrics between positions as well as between carpal, metacarpal, phalanx, and thumb fractures.

## Results

During the period of this study, a total of 304 players sustained carpal, metacarpal, phalanx, or thumb injuries, with three players having injuries in two of these categories, for a total of 307 injuries. These were classified as metacarpal (n = 110, 35.8%), carpal (n = 80, 26.1%), thumb (n = 78, 25.4%), and phalangeal (n = 39, 12.7%), as represented in Figure 1. Metacarpal injuries were significantly overrepresented, and phalanx injuries were underrepresented, in NFL players (p < 0.001, χ²). 

**Figure 1:**
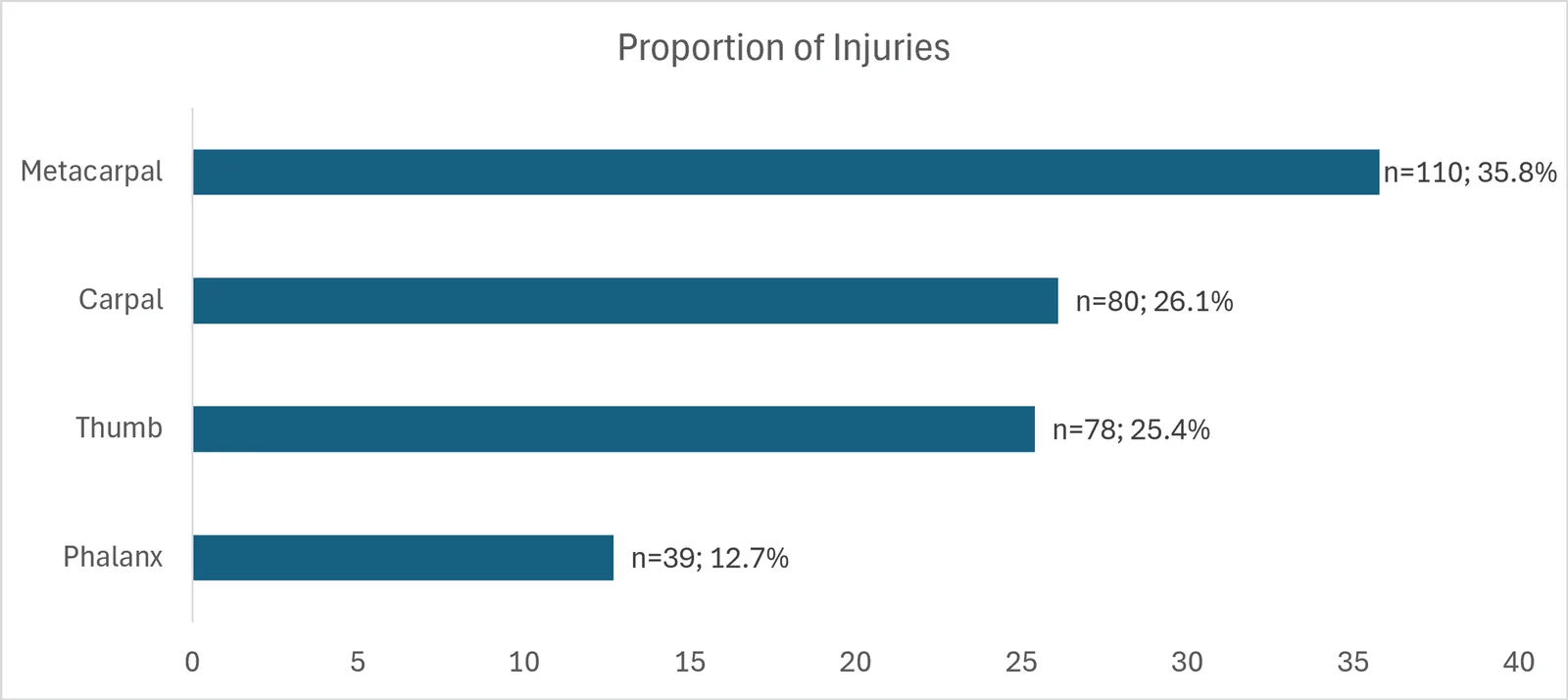
Proportion of metacarpal, carpal, phalanx, and thumb injuries. The data are presented as the number of injuries (n) and the percentage of total injuries. A p < 0.05 is considered significant (*X^2^*). Metacarpal injuries occurred most frequently, accounting for over a third of the injuries included.

Of the 304 players in this study, 15 different positions were represented, as shown in Figure 2. Linebackers (15.1%), wide receivers (12.8%), and tackles (12.5%) accounted for 40.4% of all reported hand injuries (p < 0.001, χ²). Quarterbacks (9.5%), defensive backs (9.5%), and running backs (8.6%) were also largely impacted, as shown in Figure 2. 

**Figure 2:**
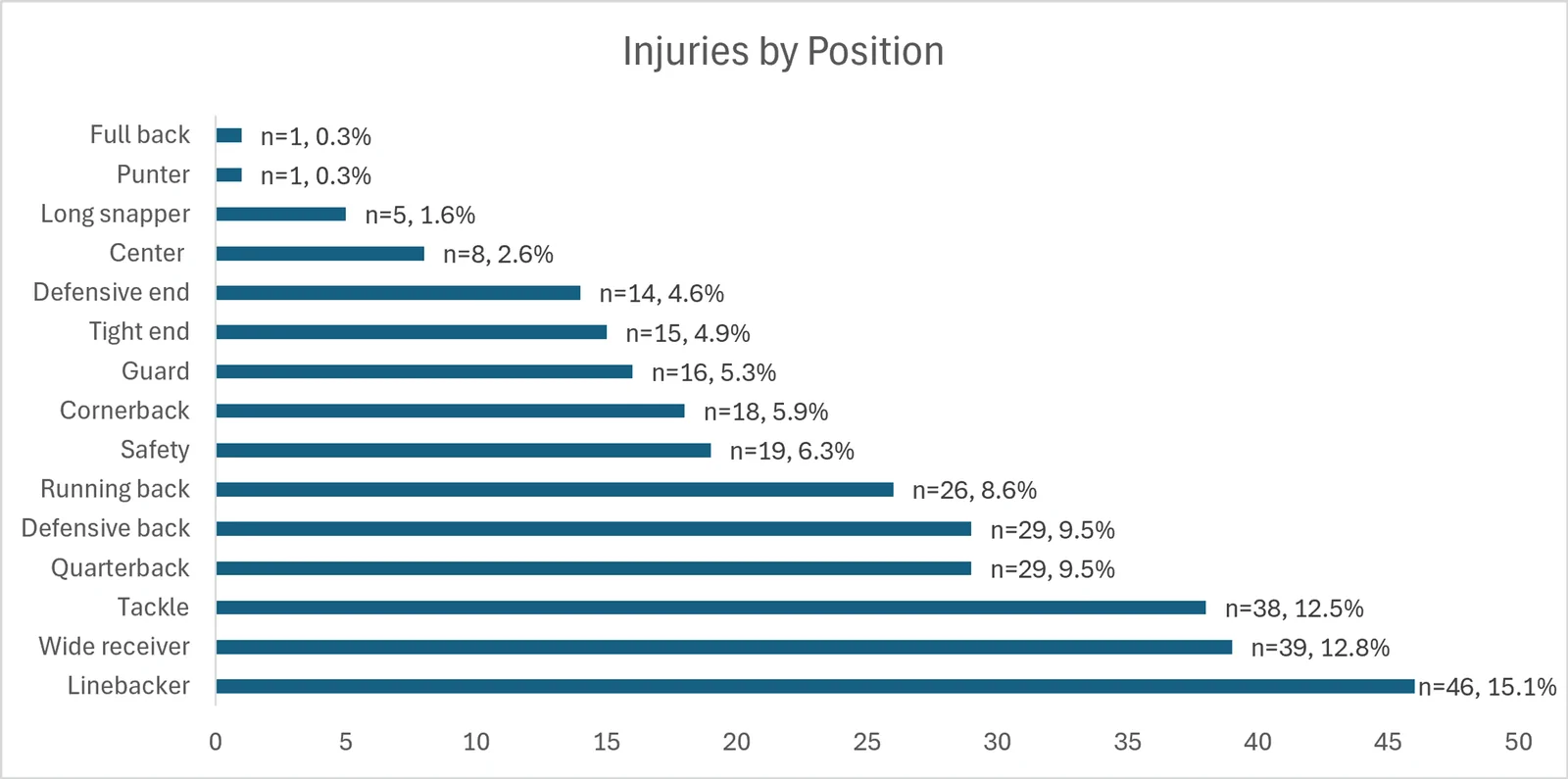
Total count of injuries for each position. The data are presented as numbers (n) and percentages of injuries for each position. A p < 0.05 is considered significant (*X^2^*). The greatest number of hand injuries were sustained by linebackers, followed by wide receivers and tackles.

Hand injuries occurred throughout the season, including the preseason and playoff games. Eighty-nine injuries occurred in September, which was statistically significant (p < 0.001, χ²), and over a third of these injuries occurred during preseason games or in September (39.5%), at the start of the season (Figure 3). 

**Figure 3:**
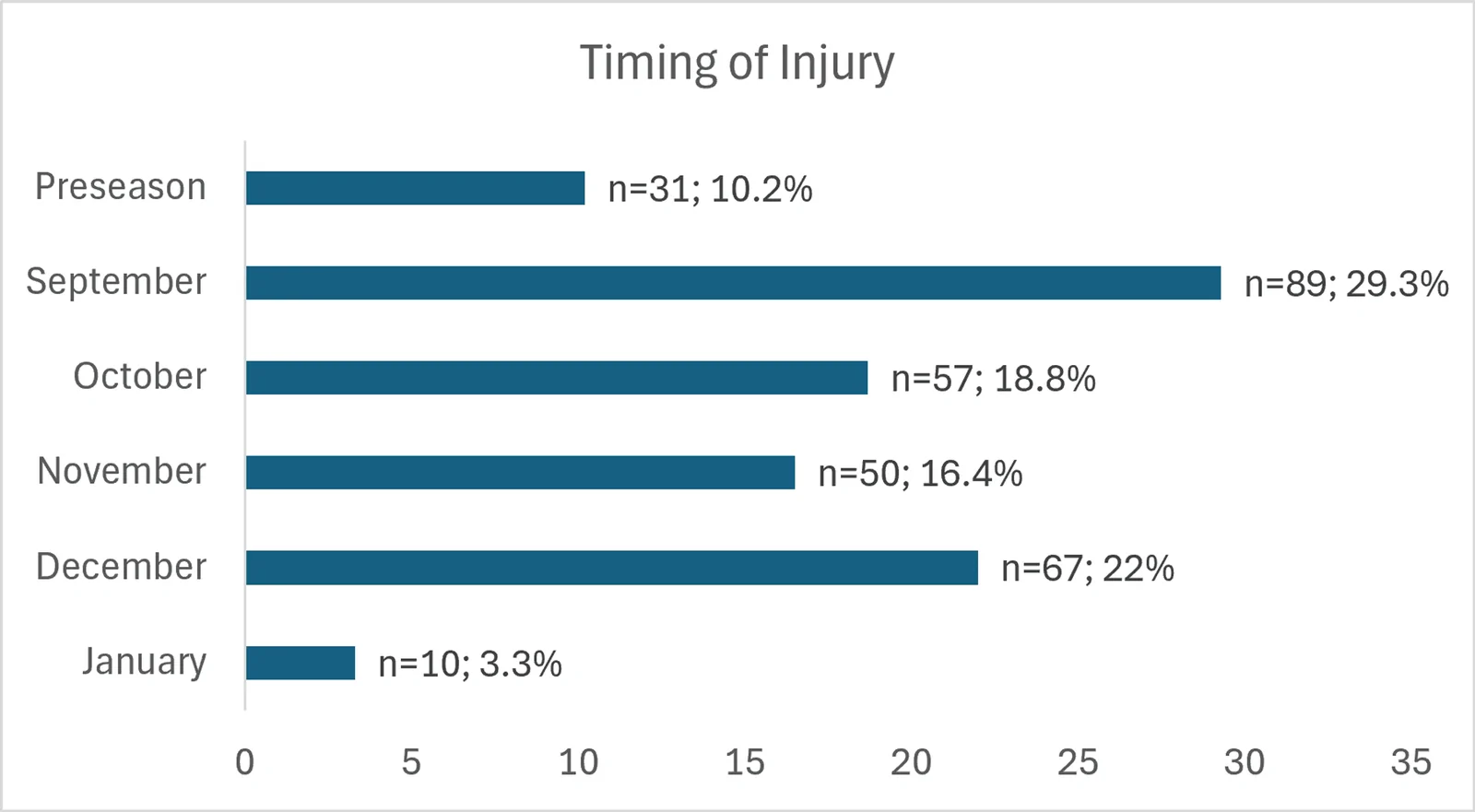
Timing of injury. The data are presented as the number of injuries (n) and the percentage of total injuries. A p < 0.05 is considered significant (*X^2^*). Most hand injuries occurred during September, at the start of the NFL season.

Fifty-five players were injured in their rookie season (18.1%), 48 were injured in their final season (15.8%), and 10 were injured in their only season in the NFL. These players were excluded from performance analysis due to a lack of pre-injury or post-injury data.

Fifty-eight of the 304 players (19.1%) did not return to the NFL after the reported hand injury. There was an association between some positions and the likelihood of returning to play after injury. Of the running backs included in this study, 30.8% did not play in the season after injury. Other positions that had a large proportion of injuries in their final season included safeties (26.3%), linebackers (22.2%), and quarterbacks (20.7%).

Certain positions were disproportionately impacted by an upper extremity injury in their rookie season as well. Of the wide receivers in this study, 30.8% sustained a hand injury in their first NFL season.

The average number of games missed due to an injury was 5.4 games; by injury, phalangeal: 5.8, carpal: 6.4, thumb: 5.4, and metacarpal: 4.30 (p = 0.02, χ²). The average decrease in games played from the preceding season to the following season was 1.02, with no significant difference when stratified by injury. The average decrease in games started was 0.5 games, with thumb injuries having the least impact, as games started increased by an average of 2.0 games (p = 0.08, χ²). The overall average change in snap counts was -31.4; by injury, phalangeal: -120.1, carpal: -20.0, thumb: -75.1, and metacarpal: +16.3 (Table 1). Safeties had the greatest decrease in games played, with an average of 1.1 fewer games, as well as the greatest decrease in games started, with an average of 2.2 fewer starts after injury. Tackles had the largest decline in snap counts, with an average of 178.1 fewer snaps after injury (Table 2). 

**Table 1:** Return to sport and performance data by injury. The data are presented as the mean for each injury type. A p < 0.05 is considered significant (*X^2^*). Metacarpal injuries had significantly less impact on games missed when compared to other injuries (p=0.02, *X^2^*). There was no significant difference in the impact on performance in the seasons following injury (games played, starts, or snap counts), and all increased on average.

Injury	Average games missed	Average change in games played	Average change in games started	Average change in snap counts
Phalanx	5.8	+1.1	+1.9	+120.1
Thumb	5.4	+1.2	+2.1	+125.7
Metacarpal	4.3	+0.8	-0.5	+50.0
Carpal	6.4	+1.1	-0.3	+31.5
Total	5.5	+1.0	+0.5	+72.8

**Table 2:** Return to sport and performance data by player position. The data are presented as the mean for each position. A p < 0.05 is considered significant (*X^2^*). Positions with fewer than 5 injuries are excluded from this table (Fullback and Punter). Quarterbacks missed the most games due to injury. However, players in this position had the greatest increase in games played, games started, and snap counts in the season following injury.

Position	Average games missed	Average change in games played	Average change in games started	Average change in snap counts
Long snapper	7.2	-2.0	0.0	-6.0
Center	4.9	-0.8	-0.3	-9.0
Defensive end	5.6	+0.2	0.0	-14.9
Tight end	5.1	+2.4	+0.5	+130.4
Guard	4.9	+4.0	+3.0	+159.1
Cornerback	4.2	+0.5	+0.2	+46.9
Safety	5.2	-0.8	-2.0	-94.8
Running back	5.7	-0.07	-0.5	-22.5
Defensive back	6.1	+0.4	+0.9	+209.3
Quarterback	7.5	+3.7	+3.4	+243.1
Tackle (O + D)	4.9	+1.0	-0.4	-41.9
Wide receiver	5.4	+1.8	+1.6	+151.2
Linebacker	4.7	+0.9	-0.2	+66.1
Total	5.5	+1.0	+0.5	+72.8

## Discussion

This study provides a comprehensive analysis of hand injuries in the NFL over 10 seasons, examining return-to-sport timelines and post-injury performance for different types of injuries across all positions. There was much variation in the incidence of carpal, metacarpal, phalanx, and thumb injuries based on player position.

A large percentage of the injuries occurred during the preseason or in September, which may suggest that players are more likely to suffer a hand injury when coming out of the offseason. This is consistent with findings summarized in previous literature. In a 2026 study by Worley et al. examining injuries in an NCAA (National Collegiate Athletic Association) Division I football team, a similar pattern in the timing of injury was identified [9]. They found the overall rate of injury for the team to be 6.52 per 1000 athletic exposures (AEs), defined as any practice, game, or training session in which a player participated. The preseason rate was much higher at 12.34 per 1000 AEs, closely followed by the fall season with 11.48. Winter, spring, and summer offseason rates were far below the mean. In another study examining lower extremity strains in NFL players over a five-year period, 1,076 of 4,015 (27%) occurred during preseason practices [10]. Athletes and trainers should take this into consideration when preparing for a new season to begin.

Of the players in this study, 19.1% did not return to the NFL after their reported hand injury. There is no way to determine whether these situations were solely due to career-ending injuries or whether other factors led to the players' retirements. Running backs, safeties, and linebackers were least likely to return after a hand injury. The demands of these positions require intact upper extremity strength and motion. Physicians and trainers should seek to optimize the management of these players to give them the best chance of returning to sport.

Linebackers, wide receivers, and tackles sustained a significantly higher proportion of hand injuries compared with other positions. The demands of these positions place these players in situations where a carpal, metacarpal, phalanx, or thumb injury is likely to occur. Although quarterbacks missed the most games compared with other positions, they also demonstrated the largest increase in games started as well as snap counts in the season after a hand injury. This pattern may suggest that physicians and trainers take a more conservative approach when treating quarterbacks with hand injuries, but these players often recover well. Additionally, guards showed the largest increase in games played and a substantial snap count increase after injury, suggesting robust return to play in these athletes. Safeties appear to suffer most from hand injuries, with an average of over five games missed. They also had the largest decline in games started and snap counts, suggesting a negative impact on post-injury performance. The aforementioned patterns should be further explored in future studies to determine if causal relationships exist between player positions and the impact of injury.

The findings of this study offer several clinical implications that may help guide providers in treating hand injuries in NFL players. The management of these athletes requires the difficult task of balancing the competing demands of a quick return to play with preservation of function and performance. The data presented in this study may help to set realistic expectations and guide shared decision-making between the athlete and treatment team.

Limitations of this study include its retrospective observational database design, which makes it difficult to account for confounding variables and to establish a causal relationship between hand injury and performance outcomes. Whether surgical treatment was pursued was inconsistently reported and thus excluded from our analysis. Future studies might explore a way to obtain this information to provide a valuable data point.

## Conclusions

Phalanx, thumb, metacarpal, and carpal fractures are common injuries in NFL players, with an average of about 25 recorded per season, and they have a measurable overall impact on player performance. Players missed an average of more than five games due to these injuries, which is nearly a third of the regular NFL season. Fifty-eight players did not return to the NFL, suggesting that hand injuries may be costly to both players and organizations and should be carefully managed by trainers and medical staff. It is important to note that there is no way to determine whether these players did not return to the NFL solely due to the injury or due to other circumstances. There was, on average, an overall improvement in performance among the players who returned the following season, which varied greatly depending on the position of the player. This study should help guide team physicians and trainers when evaluating the return-to-play timeline and anticipating the impact on players after a hand injury based on their position.
